# The relationship between right-to-left shunt and brain white matter lesions in Japanese patients with migraine: a single center study

**DOI:** 10.1186/s10194-016-0714-x

**Published:** 2017-01-07

**Authors:** Akio Iwasaki, Keisuke Suzuki, Hidehiro Takekawa, Ryotaro Takashima, Ayano Suzuki, Shiho Suzuki, Koichi Hirata

**Affiliations:** 1 Stroke Division, Department of Neurology, Dokkyo Medical University, 880 Kitakobayashi, Mibu, Shimotsuga-gun, Tochigi, 322-0293 Japan; 2 Department of Neurology, Dokkyo Medical University, Tochigi, Japan; 3 Center of Medical Ultrasonics, Dokkyo Medical University, Tochigi, Japan

**Keywords:** Migraine, Right-to-left shunt, Brain white matter lesions, Patent foramen ovale, Pulmonary arteriovenous malformations, Transcranial ultrasonography

## Abstract

**Background:**

There may be a link between right-to-left shunt (RLs) and brain white matter lesions (WMLs) in patients with migraine. In this study, we assessed the relationship between WMLs and RLs in Japanese migraine patients.

**Methods:**

A total of 107 consecutive patients with migraine with (MA) and without aura (MWOA) were included in this study. Contrast transcranial Doppler ultrasound was used to detect RLs. WMLs were graded using brain magnetic resonance imaging based on well-established criteria.

**Findings:**

The prevalence of RLs was significantly increased in the WMLs positive group (*n* = 24) compared with the WMLs negative group (*n* = 83) (75.0% vs. 47.0%, *p* = 0.015). In prevalence of WMLs between MA and MWOA patients, there were no statistical differences (*p* = 0.410). Logistic regression analysis adjusted by age and disease duration of migraine identified an RLs-positive status as the sole determinant for the presence of WMLs (OR = 6.15; 95% CI 1.82–20.8; *p* = 0.003)

**Conclusion:**

Our study suggests a possible link between RLs and WMLs in Japanese patients with migraine.

## Background

Patients with migraine have been reported to exhibit a two- to fourfold increase in WMLs compared to subjects without migraine [1, 2]. Igarashi et al. [3] reported a significantly increased prevalence of WMLs in patients with migraine compared to controls.

Also, a significant relationship between PFO and migraine has been reported [4]. In a recent meta-analysis, PFO is associated with 2.5-fold increased prevalence for migraine and 3.4-fold increased prevalence migraine with aura [5]. In a cross sectional study, we have found increased prevalence of right-to-left shunts (RLs) (62.9% vs. 44.0%) and PFO (54.8% vs. 30.0%) in patients with migraine with aura (MA) compared with those with migraine without aura (MWOA) in Asian populations [6]. Although the exact mechanisms of association between migraine and PFO remain unclear, activation of the trigeminal nerve and cerebrovascular system by the passage of metabolic substances or subclinical emboli through a PFO has been implicated [7]. In addition, several reports have described a positive relationship between RLs and brain white matter lesions (WMLs) [8]. Both PFO and pulmonary arteriovenous malformation (pAVM) are known risk factors for cryptogenic ischemic stroke in young subjects. Furthermore, elderly individuals with severe WMLs are at risk for developing stroke [9], and migraine has also been associated with an increased risk of stroke [10].

These observations suggest a possible link between migraine, RLs and WMLs. However, the association between RLs and WMLs has not been well studied in Asian populations.

We conducted a single-center, cross-sectional study consisting of consecutive migraine patients from a headache outpatient clinic to test our hypothesis that the presence of WMLs in migraine patients is associated with RLs.

## Methods

Between April 2014 and May 2016, migraine patients were recruited from our headache outpatient clinic at the Department of Neurology of Dokkyo Medical University Hospital. In Japan, unlike in Europe and America, there is no formal system of medical referral. A referral is recommended but not necessary: in fact, most of the patients who visited to our headache outpatient clinic were not referral cases [11]. MA and migraine without aura (MWOA) were diagnosed by headache specialists (RT, SS and KH) according to the International Classification of Headache Disorders, 3rd edition (beta version) [12]. Clinical information, including smoking; onset age of migraine; family history of migraine; sensitivity to light, sound or smell; and comorbid diseases such as hypertension, dyslipidemia, diabetes mellitus, and atrial fibrillation was obtained by questioning the patients. No patient had atrial fibrillation. Clinical characteristics and WMLs were compared between RLs-positive and RLs-negative groups.

RLs was assessed in all the patients by transcranial Doppler ultrasound (TCD) (Pioneer TC8080 System, Nicolet Vascular, TCD system, Tokyo, Japan) with intravenous injection of agitated saline with microbubbles by trained neurologists (AI, HT and AS). The details of measurements and settings were described elsewhere [6].

At first, prior to intravenous injection of contrast agent, simple observation was performed for 20 min. Then, contrast agent was injected intravenously during the Valsalva maneuver. If high intensity transient signals (HITS) were detected after 10 s Valsalva load release, PFO was diagnosed [13–15]. If HITS were detected without Valsalva load, PFO or pAVM was diagnosed. Large shunts were defined as greater than 26 HITS, and middle shunts were defined as 5–26 HITS. The same procedure was repeated 3 times for all the patients.

**Fig. 1:**
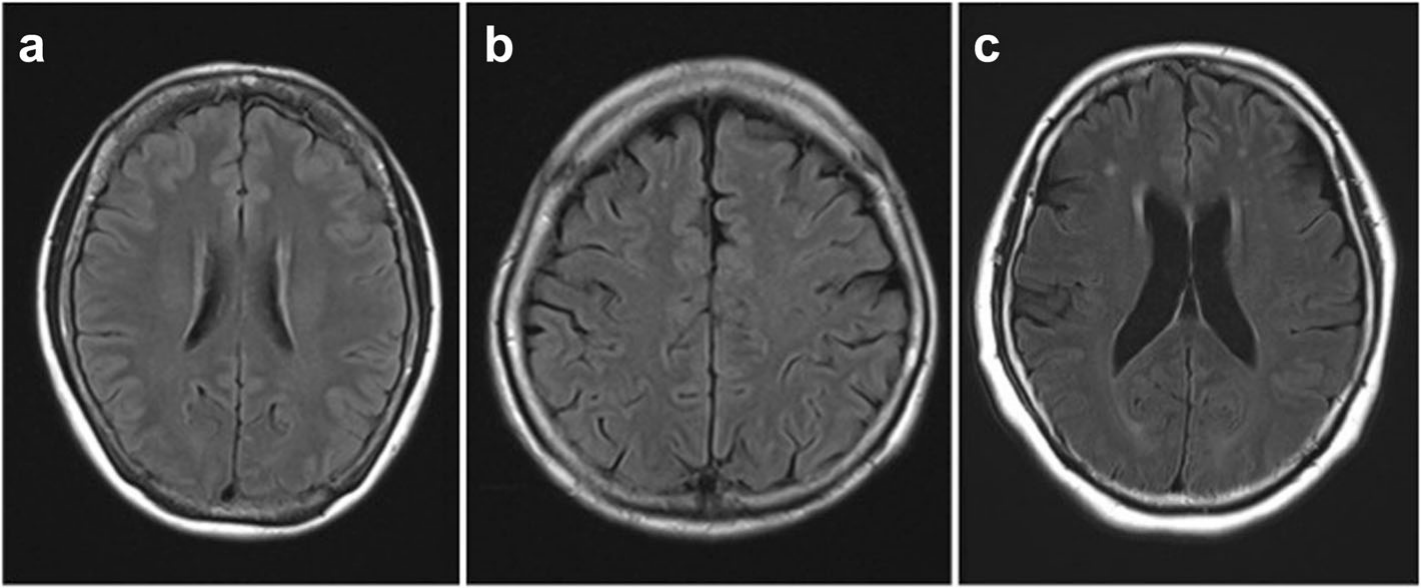
Grading of brain *white* matter lesions. **a**, grade 0 (absent); **b** grade 1 (<3 mm lesions, boundary sharp); and **c**, grade 2 (>3 mm lesions)

Patients underwent 1.5-tesla brain magnetic resonance imaging (MRI) (Symphony, Sonata, Siemens Japan Company, Tokyo, Japan). Fluid-attenuated inversion recovery (FLAIR) images (TR = 10,000 msec, TE = 98 msec, IT = 2,500 msec) with the axial or frontal section images of 5 mm slices were obtained to evaluate the extent of WMLs in the deep white matter. WMLs were defined as lesions in the deep or subcortical white matters with faint high signals on T2-weighted images, clearly high intensity on FLAIR images and iso-intensity or slightly low signals on T1-weighted signals that were distinguishable from perivascular spaces. Two trained neurologists (HT and KS) who were blinded to clinical information, including the presence of RLs, graded brain WMLs according to a previously published grading score [16]. Briefly, WMLs were classified into the following five grades: Grade 0, absent; Grade 1, <3 mm lesions, boundary sharp; Grade 2, >3 mm lesions; Grade 3, confluent foci on deep white matter; Grade 4, confluence widely distributed throughout most of the white matter. The presence of WMLs was defined as Grade 2 or greater in this study. Representative MRI images of patients are shown in Fig. 1.

### Statistical analysis

All data are described as proportions (%) and medians (range) or means (±SD). The patients were classified into the WML-positive or WML-negative group. Univariate analyses were conducted to compare characteristics between the two groups. The chi-square test and Mann–Whitney *U* test were used to compare characteristics between the WML-positive group and WML-negative groups. Based on the results, logistic regression was conducted to evaluated contributing factors to WMLs positive. All *p* values were two-tailed, and values <0.05 were considered significant. All statistical analysis was performed using IBM SPSS® for Mac, version 23 (Tokyo, Japan).

The institutional review board of Dokkyo Medical University Hospital approved the study. All patients provided written informed consent to participate.

## Findings

**Fig. 2:**
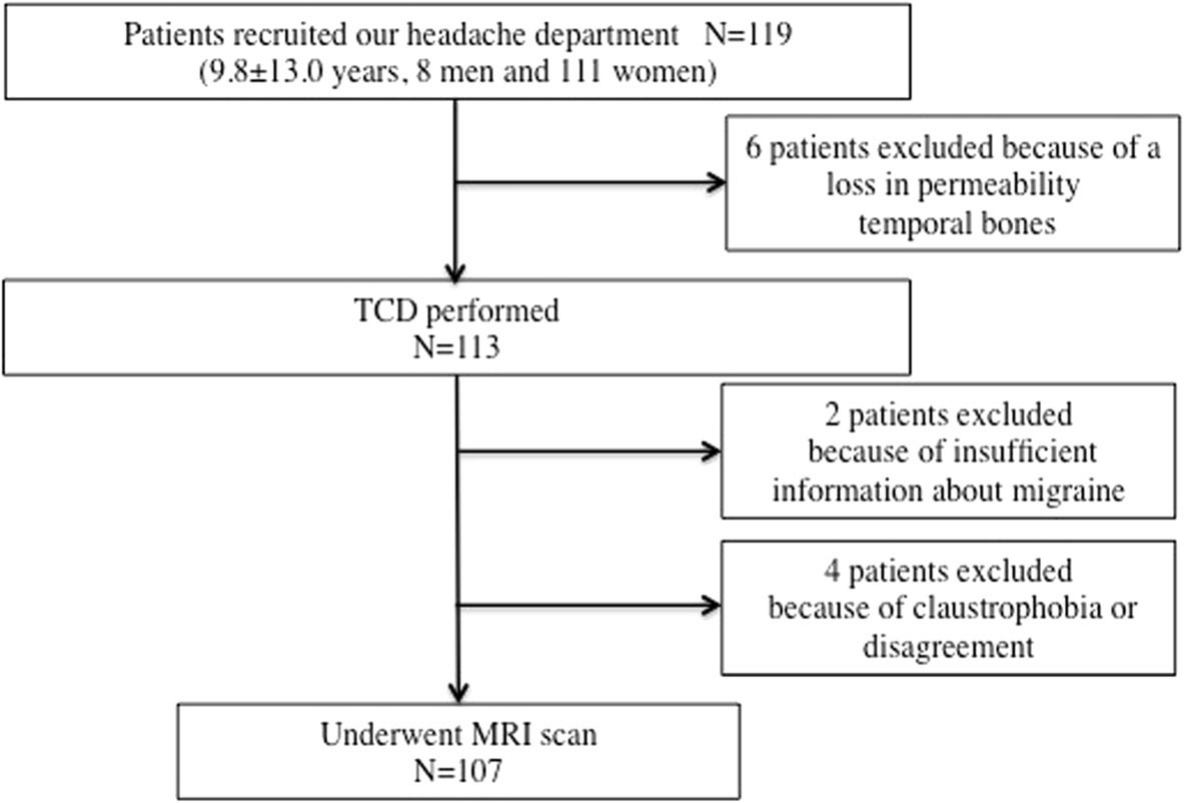
Flow chart of patient enrolment

Total 119 consecutive migraine patients (39.8 ± 13.0 years, 8 men and 111 women) were enrolled. Twelve patients were excluded from the study: 6 because of a loss in permeability during TCD evaluation of temporal bones, 2 due to insufficient information about migraine and 4 in whom MRI scans were not feasible because of claustrophobia or disagreement. Finally, 107 patients (95.5%) were included in this study (Fig. 2). Ten patients (9.3%) were diagnosed with chronic migraine (headache frequency of 15 or more days per month for more than 3 months). Of the total patients, 49 patients (45.8%) used preventive medicine for migraine.

### Detection of RLs

A total of 107 subjects (women 101, median age 39.0 years, range 14–74 years) underwent TCD examination (Table 1). When contrast agent injection with the Valsalva maneuver was performed, HITs were detected in 57 subjects (53.3%). With contrast agent administration at rest, HITs were detected in 11 subjects (10.7%). Based on these results, a total of 46 subjects (43.0%) were diagnosed with PFO and 11 subjects (10.7%) were diagnosed with PFO or pAVM. The maximum numbers of HITs per single examination were as follows: 1–5 HITs, 37 subjects; 5–26 HITs, 7 subjects; greater than 26 HITs, 13 subjects.

**Table 1:** Prevalence of RLs and patient characteristics

Characteristics	Total (*n* = 107)
RLs positive, n (%)	57 (53.3)
PFO, n (%)	46 (43.0)
PFO or pulmonary arteriovenous malformations suspected, n (%)	11 (10.3)
Large shunt, n (%)	13 (12.1)
Age, median (range) years	39.0 (14–74)
Sex, male, n (%)	6 (5.6)
Aura, n (%)	59 (55.1)
Hypertension, n (%)	8 (7.5)
Diabetes mellitus, n (%)	3 (2.8)
Dyslipidemia, n (%)	11 (10.3)
Smoking, n (%)	7 (6.5)
Family history of migraine, n (%)	71 (66.4)
Photophobia, n (%)	81 (75.7)
Phonophobia, n (%)	79 (73.8)
Hypersensitivity to smell, n (%)	60 (56.1)
Altered taste, n (%)	11 (10.3)
Allodynia, n (%)	7 (6.5)
Onset age of migraine, median (range) years	18.0 (9–40)

*MA* Migraine with aura, *MWOA* Migraine without aura, *RLs* Right to left shunts

Chi-square test and Mann–Whitney *U* test

### Comparison of factors between WML-positive and WML-negative patients

Of 107 subjects, 24 patients (22.4%) had brain WMLs, all of which were grade 2. No one exhibited grade 3 or 4 WMLs. There was no statistical difference in the severity of WMLs between patients with MWA and MWOA. None of chronic migraine patients had WMLs. The rate of preventive medicine use did not differ between the WML-positive and WML-negative groups (*p* = 0.997).

**Fig. 3:**
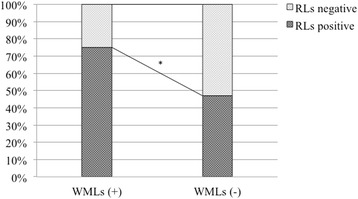
Prevalence of RLs between WML-positive group and WMLs-negative group. *p = 0.015. RLs: Right to left shunts, WMLs: White matter lesions

The prevalence of RLs and PFO in the WML-positive group was significantly higher than that in the WML-negative group (RLs, 75.0% vs 47.0%, *p* = 0.015; PFO, 62.5% vs 37.3%, *p* = 0.028) (Fig. 3). Regarding the ratio of patients with MA or MWOA, there was no differences prevalence between the WML-positive group and WML-negative groups (*p* = 0.410). The WML-positive group was significantly older than the WML-negative group (*p* < 0.001). Disease duration for migraine was also longer in the WML-positive group (median: 29.5 years) than the WML-negative group (median: 17.0 years) (*p* = 0.001). With the exception of RLs, age and disease duration, there were no differences in characteristics between the WML-positive and WML-negative groups (Table 2).

**Table 2:** Comparison of characteristics between WMLs positive and negative group

Characteristics	WMLs (+) (*n* = 24)	WMLs (−) (*n* = 83)	*p* value
RLs positive, n (%)	18 (75.0)	39 (47.0)	*0.015*
PFO, n (%)	15 (62.5)	31 (37.3)	*0.028*
Large shunt, n (%)	5 (20.8)	8 (9.6)	0.139
Age, median (range), years	46.5 (24–74)	36.0 (14–65)	*<0.001*
Sex, male, n (%)	0 (0)	6 (7.2)	0.175
Migraine with aura (MA), n (%)	15 (62.5)	44 (53.0)	0.410
Hypertension, n (%)	2 (8.3)	6 (7.3)	0.856
Diabetes mellitus, n (%)	1 (4.2)	2 (2.4)	0.646
Dyslipidemia, n (%)	2 (8.3)	9 (10.8)	0.721
Smoking, n (%)	1 (4.2)	6 (7.2)	0.593
Family history of migraine, n (%)	16 (66.7)	55 (66.3)	0.971
Photophobia, n (%)	19 (79.2)	62 (74.7)	0.653
Phonophobia, n (%)	18 (75.0)	61 (73.5)	0.882
Hypersensitivity to smell, n (%)	15 (62.5)	45 (54.2)	0.471
Altered taste, n (%)	5 (20.8)	6 (7.2)	0.053
Allodynia, n (%)	2 (8.3)	5 (6.0)	0.687
Duration of migraine, median (range) years	29.5 (0–52)	17.0 (0–51)	*0.001*
Onset age of migraine, median (range), years	17.5 (9–37)	18.0 (4–40)	0.625

*MA* Migraine with aura, *MWOA* Migraine without aura, *RLs* right-to-left shunt, *WMLs* white matter lesions, *NS* not significant

Chi-square test and Mann–Whitney *U* test

<0.05 is significantly italicize

Based on the results of univariate analysis, multivariate analysis was conducted to adjust for age and disease duration of migraine that may have influenced WML prevalence. Multivariate logistic regression analysis indicated that the presence of RLs were independently associated with the presence of WMLs (*p* = 0.003; OR = 6.15; 95% CI 1.82–20.8).

## Discussion

In our study, we investigated the prevalence of RLs and the relationship between clinical background factors, type of migraine and WMLs.

An increased prevalence in WMLs in the RLs-positive group compared to the RLs-negative group was observed. Furthermore, the presence of RLs was an independent predictor for the presence of WMLs in multivariate regression analysis. Aging and vascular risk factors, such as hypertension, diabetes mellitus, and dyslipidemia, have been associated with WMLs in individuals without migraine [17–19]; however, these findings were not supported by our study. Our findings suggest a significant relationship between RLs and WMLs in Japanese patients with migraine. Several studies have reported an increased prevalence of WMLs in patients with migraine compared to individuals without headaches after controlling for vascular factors [1–3, 20]. In a study by Igarashi et al. [3], an increased prevalence of WMLs was found in Japanese patients with migraine, but WMLs were not associated with headache severity or disease duration. In our study, none of chronic migraine patients had WMLs, suggesting headache frequency does not seem to be an important factor for WMLs.

Several studies showed migraine, especially migraine with aura to be a risk factor for WMLs [21, 22]. Additionally, the risk for ischemic stroke was reported to be higher in patients with migraine with aura than with migraine without aura [23]. In contrast, other studies did not show differences in prevalence of WMLs between MWA and MWOA patients [24]. Zhang et al. [25] described that, although no differences were observed in WMLs in MA and MWOA groups, substantially lower cerebral blood flow found in association with high WMLs load suggests that WMLs in MWA may be associated with alteration in resting cerebral blood flow. In our study, we did not observe any differences in WMLs between MA and MWOA groups. However, it is possible that other factors, such as L-arginine, dimethylarginine levels [24] and D-dimer levels as well as headache attack frequencies [26], might have affected WMLs independent of the presence or absence of auras.

Several studies analyzing cohorts of Caucasian patients with migraine did not show a significant association between RLs and WMLs [27, 28]. In contrast, Park et al. [29] showed that the presence of RLs is an independent predictor for the prevalence of small, but not medium or large, WMLs based on multivariate analysis of 425 subjects (242 patients with migraine and 183 patients with tension-type headache). The authors speculated that small paradoxical emboli might have caused small WMLs, triggering migraine headaches. In agreement with the referenced study, our current study showed a significant increase in WMLs prevalence in the RLs-positive group compared with the RLs-negative group, supporting a possible link between RLs and WMLs in migraine. Our results and those from Park et al.’s study [29] suggest that RLs may be associated with small, but not large, WMLs in Asian patients with migraine.

Although the pathogenesis associated with WMLs in patients with migraine is unclear, subclinical ischemia, blood brain barrier permeability, reactive astrocytic gliosis-induced injury and endothelial dysfunction may have a role, as well as RLs [4, 20]. Regarding the mechanism underlying the potential relationship between PFO and migraine, PFO may allow vasoactive chemicals, such as serotonin and endothelin, or embolic material to bypass the pulmonary filter and reach the cerebral circulation to induce a migraine attack [30, 31]. Additionally, paradoxical air microemboli through the PFO may induce cerebral electrical activity, triggering migraine attacks [32]. In addition, these vasoactive chemicals may play important role in increased WMLs. Several studies found that migraine is associated to several vascular disorders, such as stroke or ischemic heart diseases [33]. In some cases, repeated small paradoxical embolisms may result in an increase in WMLs.

The current study has several limitations. First, not all patients with migraine who were seen in our hospital underwent TCD examination and MRI scanning; therefore, selection bias might have affected the results. The male to female ratio was lower in our study (1:16) than that in a population-based study of migraine 191 patients in Japan (1:3.6) [34], which may have caused by selection bias. Because of small sample size of male patients, we could not evaluate difference in characteristics including WMLs between male and female migraine patients.

Second, embolic risk factors, such as D-dimer levels, use of oral contraceptives and past history of pregnancy and recurrent miscarriage were not evaluated. Oral contraceptives are well known as a risk factor of stroke in migraine and non-migraine subjects [35]. According to a study of 146 parous and nulliparous women [36], history of pregnancy was not related to the presence of WMLs; however, PFO was not evaluated. Thus, further evaluation of these factors is needed. These factors might have affected the prevalence of WMLs due to silent paradoxical cerebral infarctions.

## Conclusions

RLs were found in over half of a cohort of Japanese patients with migraine. Our study suggests a possible link between RLs and WMLs in Japanese migraine patients.

## Abbreviations

FLAIR: Fluid-attenuated inversion recovery
HITS: High-intensity transient signals
MA: Migraine with aura
MCA: Middle cerebral artery
MRI: Magnetic resonance imaging
MWOA: Migraine without aura
pAVM: pulmonary arteriovenous malformation
PFO: Patent foramen ovale
RLs: Right to left shunts
TCD: transcranial Doppler
WMLs: White matter lesions
